# Co‐development of a school‐based and primary care‐based multicomponent intervention to improve HPV vaccine coverage amongst French adolescents (the PrevHPV Study)

**DOI:** 10.1111/hex.13778

**Published:** 2023-06-13

**Authors:** Aurélie Bocquier, Sébastien Bruel, Morgane Michel, Anne‐Sophie Le Duc‐Banaszuk, Stéphanie Bonnay, Marion Branchereau, Karine Chevreul, Sandra Chyderiotis, Aurélie Gauchet, Bruno Giraudeau, Dragos‐Paul Hagiu, Judith E. Mueller, Amandine Gagneux‐Brunon, Nathalie Thilly, PrevHPV Study Group

**Affiliations:** ^1^ APEMAC Université de Lorraine Nancy France; ^2^ Department of General Practice, Jacques Lisfranc Faculty of Medicine Saint‐Etienne‐Lyon University Saint‐Etienne France; ^3^ Health, Systemic, Process UR 4129 Research Unit, University Claude Bernard University of Lyon Lyon France; ^4^ ECEVE UMR 1123, Université de Paris Cité Paris France; ^5^ Assistance Publique‐Hôpitaux de Paris, Hôtel Dieu, URC Eco Ile‐de‐France/Hôpital Robert Debré Unité d'épidémiologie clinique Paris France; ^6^ Centre Régional de Coordination des Dépistages des cancers‐Pays de la Loire Angers France; ^7^ Emerging Disease Epidemiology Unit, Institut Pasteur Université Paris Cité Paris France; ^8^ LIP/PC2S Université Grenoble Alpes Grenoble France; ^9^ LIP/PC2S Université Savoie Mont Blanc Chambéry France; ^10^ SPHERE U1246, Université de Tours, Université de Nantes INSERM Tours France; ^11^ INSERM CIC 1415 CHRU de Tours Tours France; ^12^ CIC‐INSERM 1408, CHU de Saint‐Etienne France; ^13^ Univ. Rennes, EHESP, CNRS, Inserm, Arènes ‐ UMR 6051 RSMS (Recherche sur les Services et Management en Santé) ‐ U 1309 Rennes France; ^14^ Centre International de Recherche en Infectiologie, Team GIMAP, Univ Lyon, Université Jean Monnet, Université Claude Bernard Lyon 1, Inserm, U1111, CNRS, UMR530, CIC INSERM 1408 Vaccinologie CHU de Saint‐Etienne Saint‐Etienne France; ^15^ Département Méthodologie, Promotion, Investigation Université de Lorraine, CHRU‐Nancy Nancy France

**Keywords:** co‐construction, complex Intervention, eHealth tools, human papillomavirus, motivational interview, vaccination behaviours

## Abstract

**Introduction:**

Despite various efforts to improve human papillomavirus (HPV) vaccine coverage in France, it has always been lower than in most other high‐income countries. The health authorities launched in 2018 the national PrevHPV research programme to (1) co‐develop with stakeholders and (2) evaluate the impact of a multicomponent complex intervention aimed at improving HPV vaccine coverage amongst French adolescents.

**Objective:**

To describe the development process of the PrevHPV intervention using the GUIDance for rEporting of intervention Development framework as a guide.

**Methods:**

To develop the intervention, we used findings from (1) published evidence on effective strategies to improve vaccination uptake and on theoretical frameworks of health behaviour change; (2) primary data on target populations' knowledge, beliefs, attitudes, preferences, behaviours and practices as well as the facilitators and barriers to HPV vaccination collected as part of the PrevHPV Programme and (3) the advice of working groups involving stakeholders in a participatory approach. We paid attention to developing an intervention that would maximise reach, adoption, implementation and maintenance in real‐world contexts.

**Results:**

We co‐developed three components: (1) adolescents' and parents' education and motivation using eHealth tools (web conferences, videos, and a serious video game) and participatory learning at school; (2) general practitioners' e‐learning training on HPV using motivational interviewing techniques and provision of a decision aid tool and (3) easier access to vaccination through vaccination days organised on participating middle schools' premises to propose free of charge initiation of the HPV vaccination.

**Conclusion:**

We co‐developed a multicomponent intervention that addresses a range of barriers and enablers of HPV vaccination. The next step is to build on the results of its evaluation to refine it before scaling it up if proven efficient. If so, it will add to the small number of multicomponent interventions aimed at improving HPV vaccination worldwide.

**Patient or Public Contribution:**

The public (adolescents, their parents, school staff and health professionals) participated in the needs assessment using a mixed methods approach. The public was also involved in the components' development process to generate ideas about potential activities/tools, critically revise the successive versions of the tools and provide advice about the intervention practicalities, feasibility and maintenance.

## 1 INTRODUCTION

Human papillomavirus (HPV) infection is the most common viral infection of the reproductive tract and a major public health issue.^1^, ^2^ Depending on HPV genotypes, persistent HPV infections can cause anogenital warts (HPV 6/11), precancerous lesions of the cervix, vagina, vulva, anus, penis and head and neck, which may sometimes progress to cancers.^3^ The two most common ‘high‐risk’ genotypes (HPV 16/18) cause about 70% of all cervical cancers, the most common HPV‐related cancers.^2^ It is the fourth most frequent cancer in women worldwide, accounting for 604,127 new cases and 341,831 deaths in 2020 (respectively, 3379 and 1452 in France).^4^, ^5^

Vaccination is the most effective primary prevention strategy against HPV infection.^2^, ^5^ Bivalent, quadrivalent, and nonavalent vaccines have been marketed. All vaccines target HPV 16/18, while the quadrivalent vaccine also targets HPV 6/11 and the nonavalent one adds five oncogenic types.^2^ HPV vaccination programmes have shown substantial impacts on HPV infections, anogenital warts and high‐grade precancerous cervical lesions.^6^, ^7^, ^8^, ^9^ They have also recently been associated with a reduced risk of invasive cervical cancer.^10^, ^11^ HPV vaccines have an ‘excellent safety profile’ according to the World Health Organisation.^2^

Since 2006, most high‐income countries have introduced HPV vaccination in their vaccination schedules for adolescents, either for girls only or for girls and boys, depending on the country.^12^, ^13^ In France, HPV vaccination was introduced for girls in 2007 and the nonavalent vaccine is now recommended to all adolescents aged 11–14 years. Despite various efforts by health authorities to improve HPV vaccine uptake,^14^, ^15^ complete HPV vaccine coverage has always been lower than in most other high‐income and European countries,^12^, ^13^ estimated at 23.7% amongst 16‐year girls in 2018^16^ (see details on the French context in Section 2.1).

In this context, the French Institute for Public Health Research (IReSP) and the theme‐based multiorganisation institutes for cancer and for public health (ITMO Cancer and ITMO Public Health) launched in 2018 a national research programme to improve HPV vaccine coverage amongst French adolescents (The PrevHPV Programme—https://iresp.net/presentation-du-projet-prevhpv/). This programme is conducted by a consortium of eight French research teams with expertise in epidemiology, public health, primary care, health psychology, infectious diseases, health economics and biostatistics (The PrevHPV Consortium—see list in Supporting Information Materials: Appendix A) and funded as part of the National Cancer Plan 2014–2019. The aim of the PrevHPV Programme was to (1) co‐develop with stakeholders and (2) evaluate the impact of a multicomponent complex intervention^17^ that targets several population groups and organisational levels.

The objective of the present article is to describe the development of the PrevHPV intervention. The protocol for the evaluation of its effectiveness, efficiency and implementation (NCT 04945655) has been described in detail elsewhere.^18^

## 2 METHODS

We describe the development of the PrevHPV intervention using the GUIDance for rEporting of intervention Development (GUIDED) framework as a guide^19^ (see completed GUIDED checklist in Supporting Information Materials: Appendix B). In accordance with this framework, we first describe the context in which the intervention was developed.

### 2.1 Context of the PrevHPV intervention

In France, HPV vaccination was initially recommended for girls aged 14 years,^20^ then for girls aged 11–14 years^21^; in 2021, it was included in the vaccine schedule for all adolescents, girls and boys, aged 11–14 years.^22^ The currently recommended vaccine is the latest nonavalent one with two injections 6 months apart. A catch‐up with three injections is possible up to age 19 and for men having sex with men up to age 26.

HPV vaccination in France depends on persons' initiative, requires parental authorization for those under 18 years, and is prescribed and administered by physicians or midwives; in practice, general practitioners (GPs) are the main prescribers and providers of HPV vaccination, for both doses.^23^ Since April 2022, under specific medical prescriptions, it can also be administered to individuals aged 16 or older by nurses or pharmacists trained in vaccination. There is currently no nationwide school‐based vaccination programme in France. Care pathways to access vaccination often include several steps: for the majority of cases, adolescents and their parents must first get the vaccine prescription during an appointment with a physician, then go to a community pharmacy to obtain the vaccine, and finally, make another appointment with their physician for its administration. Occasionally, individuals will also get vaccinated at vaccination centres, but their geographical accessibility can be difficult. The HPV vaccine is costly (116 euros for 1 dose in 2022). It is only partially (65%) covered by the National Social Health Insurance but the financial barrier to access remains low as 95% of the population with complementary health insurance are fully reimbursed.

France has been one of the European countries with the highest percentage of the general population with low confidence in vaccine safety for a long time, and the recent 2020 data confirmed this fact.^24^ Regarding the HPV vaccine, 32% of French mothers of adolescent girls agree that the HPV vaccine may lead to long‐term health problems and 20% that it is unsafe.^25^ This may partly result from controversies that occurred in France about HPV vaccine efficacy and safety (especially its suggested association with autoimmune diseases). Despite the accumulation of evidence that the HPV vaccine does not have severe adverse effects,^26^, ^27^, ^28^ the French medical community has been debating the benefits and risks of the HPV vaccine, including possible concurrence with the Pap screening programme. Healthcare providers have an essential role in influencing parental decisions towards HPV vaccination.^25^ Even if most of the French GPs (60%–70%) frequently recommend the HPV vaccine, some do not systematically mention the HPV vaccine with adolescents and their patients, especially GPs who are prone to vaccine hesitancy. About 25% of the GPs have doubts about HPV vaccine safety and/or efficacy and these doubts strongly influence their recommendation practices.^29^, ^30^, ^31^ And even when GPs are convinced of the importance of HPV vaccination, they may face difficulties during interactions with patients: 80% of GPs acknowledge having difficulties in informing about HPV vaccination and convincing hesitant patients to get vaccinated.^29^, ^30^, ^31^

### 2.2 Purpose of the PrevHPV intervention development process

The overall aim of the PrevHPV intervention was to improve HPV vaccine coverage amongst French adolescents. The aim of the PrevHPV intervention development process was to develop an evidence‐based and theory‐based multicomponent intervention that addresses all identified barriers to HPV vaccination in France and had the potential to be implemented in routine and spread to the whole country.

Based on the scientific literature (see Section 2.5), three components were identified: adolescents' and parents' education and motivation (component 1); GPs' training (component 2) and easier access to vaccination (component 3).

### 2.3 Target populations

Target populations of the PrevHPV intervention included:

1. adolescents attending middle schools, typically aged 11–14 years, who are the main target population for HPV vaccination in France^22^;
2. parents of adolescents attending middle school, who decide whether to vaccinate their child and
3. GPs, who prescribe most HPV vaccines in France,^23^, ^32^ and have a fundamental role in patients' decision‐making process towards vaccination.^25^, ^33^

### 2.4 Contribution of published intervention development approach

The UK Medical Research Council (MRC) framework for developing and evaluating complex interventions guided our overall approach to the development of the PrevHPV intervention. It recommends incorporating evidence and theories into the intervention development process.^17^

### 2.5 How evidence from different sources informed the intervention development process

To develop the PrevHPV intervention, we based our decisions on findings from published evidence, primary data collected as part of the PrevHPV Programme, and the advice of working groups involving stakeholders (see details in Section 2.9).

#### 2.5.1 Published evidence

*Facilitators and barriers to the uptake of HPV vaccination*: The following facilitators of HPV vaccination have been identified in systematic literature reviews: recent or regular visits with a physician, physician recommendation, parental acceptance, peer encouragement and health insurance coverage. The identified barriers included the cost of the vaccine, parental concerns (child not sexually active, safety of the vaccine, belief that the vaccine will encourage sexual activity, preference to wait till their child is older) and lack of information/knowledge.^33^, ^34^, ^35^

A meta‐analysis showed that physician recommendation had the greatest influence on parents' uptake of HPV vaccine for their child, followed by HPV vaccine safety concerns.^36^

*Interventions to improve general vaccination rates amongst adolescents*: We used the catalogue published by the European Centre for Disease Prevention and Control, which offers a collection of interventions that address vaccine hesitancy in general^37^ and other published evidence (e.g., a review of the literature on adolescent vaccination^38^).

The evidence suggests that the use of a combination of different interventions (i.e., multicomponent/multilevel interventions, each component/level addressing an identified barrier) appears to be more effective than single‐component interventions.^39^ Of note, educational strategies based on motivational interviews implemented in maternity wards have been found effective in reducing vaccine hesitancy amongst parents of newborns; it may be a promising way to motivate hesitant individuals to accept vaccination.^40^

*Interventions to improve HPV vaccination coverage*: Less evidence is available for interventions aiming to increase HPV vaccine uptake. Interventions targeted (separately or in combination) adolescents, parents, health professionals and the environment.

Interventions targeting both parent's and adolescents' psychosocial factors (knowledge, beliefs, outcome expectations, intention to vaccinate) have shown promising results. Amongst interventions targeting health professionals, those which combined reminder and education were found to be more effective. Overall, substantial impacts were observed with multicomponent/multilevel interventions combining interventions at the parental/adolescent and provider levels.^41^, ^42^, ^43^, ^44^, ^45^, ^46^

Strategies at the environmental level may take place in hospitals, postpartum units, schools and universities/community colleges.^43^ In particular, evidence shows that most European countries with high HPV vaccine coverage such as Belgium Flanders, the United Kingdom and Scandinavian countries have implemented school‐based vaccination programmes with no mandatory medical prescription.^13^

*Potential of eHealth technologies to increase vaccination rates*: An overview of systematic reviews led to a recommendation of using and evaluating eHealth technologies (i.e., information and communication technologies in support of health and health‐related areas) to encourage immunizations and increase vaccination adherence.^47^ eHealth tools (e.g., videos, websites, serious video games) are promising to improve HPV vaccine uptake.^48^, ^49^

#### 2.5.2 Primary data collected as part of the PrevHPV Programme

Before and during the development of the PrevHPV intervention, we carried out the PrevHPV diagnostic phase aimed at identifying knowledge, beliefs, attitudes, behaviours and practices, preferences, as well as the facilitators and barriers to HPV vaccination amongst four different population groups in France: adolescents, their parents, school staff (e.g., teachers, school nurses) and health professionals (GPs and health students). We also aimed at assessing the acceptability of school involvement in promoting the HPV vaccine and carrying out HPV vaccinations in schools.

We used a mixed methods approach and carried out quantitative cross‐sectional online surveys, qualitative studies using focus groups and semistructured individual interviews, and discrete choice experiment (DCE).^50^ Data were collected from January 2020 to May 2021. See Supporting Information Materials: Appendix C, for details on the number of participants in each survey/population group.

Results from this diagnostic phase informed the intervention development process, for example, those from the DCE study which used quantitative cross‐sectional online surveys to estimate preferences and pretest communication contents amongst adolescents. It showed that a statement presenting a low vaccine coverage positively (‘Already one‐third of pupils of your school have registered to get vaccinated’) was more effective than referring to insufficient coverage (‘Not enough pupils…’) to motivate vaccine acceptance (odds ratio [OR], 95% confidence interval: 1.48 [1.23, 1.78]). This was also the case of statements related to social conformism: ‘Most pupils of your school have registered to get vaccinated (80%)’ (OR: 1.98 [1.64, 2.38]) and ‘In some countries like England and Portugal, >80% of teens are vaccinated’ (OR: 1.94 [1.61, 2.35]). Prevention of cancer led to higher acceptance amongst girls compared to the prevention of genital warts, while the notion of sexual transmission had no substantial impact on either gender.^51^

### 2.6 How theory informed the intervention development process

#### 2.6.1 Theoretical frameworks

We developed the PrevHPV intervention using the Integrated Behavior Change (IBC) Model^52^ as the theoretical background. Drawing from several previous theories (e.g., the Theory of Planned Behavior,^53^ the Self‐Determination Theory^54^), the IBC Model posits autonomous motivation (i.e., a person acts because he/she is convinced that a particular behaviour is good for his/her health) as a distal determinant of behaviour. The effects of autonomous motivation on behaviour are mediated by attitudes, subjective norms and perceived behavioural control which themselves determine intention. It thus ascribes much importance to people's need for autonomy, which, in the case of vaccination, can be supported by a healthy environment (e.g., during interactions with physicians). In addition, the IBC model stresses the role of action planning as a way to reduce the gap between intention and behaviour.

#### 2.6.2 PrevHPV intervention theory

**Figure 1:**
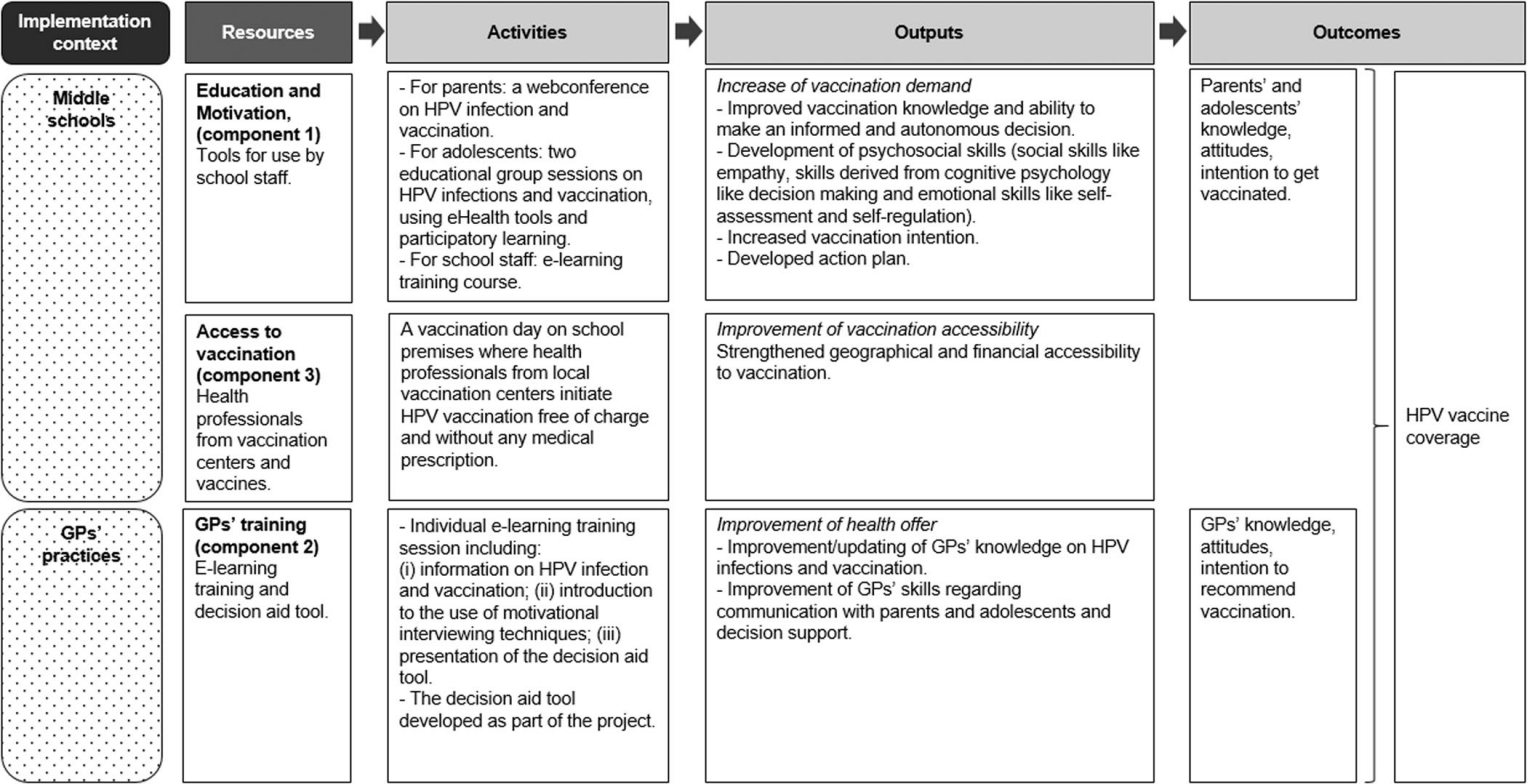
General logic model of the PrevHPV intervention. GP, general practitioner; HPV, human papillomavirus.

We developed a general logic model for the PrevHPV intervention (Figure 1) based on evidence from the literature (see Section 2.5) and the theoretical model presented above. The PrevHPV intervention comprises three components targeting the three key stakeholders involved in the HPV vaccination (adolescents, parents and health professionals):

1. Adolescents' and parents' education and motivation (component 1): it aims at increasing the vaccination demand through the development of adolescents' and parents' individual psychosocial skills, knowledge and their ability to make an informed and autonomous decision. The psychosocial skills can be divided into three categories: social skills (e.g., empathy, communication, advocacy); psychological skills from cognitive psychology (e.g., decision making) and emotional skills (e.g., self‐assessment and self‐regulation);
2. GPs' training (component 2): it aims at improving health offer, especially health professionals' recommendation for vaccination. On the one hand, it improves health professionals' knowledge of HPV and its prevention; on the other hand, it improves their skills in terms of communication with parents and adolescents using motivational interviewing techniques and decision support and
3. Easier access to vaccination (component 3): it aims at strengthening geographic and financial accessibility to vaccination by bringing the care environment into the school environment.

### 2.7 Guiding principles during the intervention development process

Our main focus was to develop an effective intervention that would maximise reach (i.e., participation of the target population), adoption (i.e., participation of practices/schools), implementation and maintenance within French middle schools and GPs' practises, in accordance with the RE‐AIM framework.^55^

For component 1 (adolescents' education and motivation), to minimize the cost of the intervention and maximize chances of maintenance, we developed activities and tools that can be implemented by regular school staff (e.g., school nurses, teachers). This includes an e‐learning training course to help these professionals develop their knowledge and skills to conduct educational group sessions on HPV infections and vaccination. Besides, to develop the content of the tools, we followed the recommendations for health education amongst young people and included various educational methods: the provision of information, active participation and development of psychosocial skills.^56^ In particular, we aimed at developing playful activities/tools to motivate the active and interactive participation of the adolescents to involve them in their learning.

Regarding component 2 (GPs' training), we took care to minimize the time required for GPs and maximize the accessibility of the training (online format) and ease of use of the decision aid tool.

For component 3 (easier access to vaccination), we aimed to develop documents (e.g., information sheets and consent forms for parents, templates of posters to inform pupils on school premises) that can be used in routine practice easily. We also facilitated the first contact between schools and vaccination centres and then let them discuss to organise the vaccination days.

### 2.8 How stakeholders contributed to the intervention development process

#### 2.8.1 Steering committee

A steering committee is in charge of supervising the progress of all aspects of the PrevHPV Programme, including the development of the multicomponent intervention, and meets once a year. It comprises the scientific leaders of the eight teams of the PrevHPV Consortium, as well as representatives of the following regional/national institutions: Inserm (French National Institute for Health and Medical Research), IReSP, ITMO Cancer AVIESAN, ITMO Public Health AVIESAN, INCa (French National Cancer Institute), Santé publique France (French Public Health Agency), Ministry of Health, Ministry of National Education and the Ile‐de‐France Regional Health Agency.

#### 2.8.2 Working groups involving stakeholders

For each component of the intervention, we set up a working group comprising members of the consortium and several professional stakeholders (e.g., school nurses, staff from vaccination centres, GPs—see details in Table 1). Each group aimed at defining the organisation of the component (e.g., activities, duration, content, the role of each actor) and developing the tools for a participatory approach in a co‐construction process.^57^ They met virtually approximatively every month throughout the development process (January 2020 to June 2021). Stakeholders generated ideas about potential activities/tools together with members of the consortium, critically revised the successive versions of the tools and provided advice about the intervention practicalities, feasibility and maintenance.

**Table 1:** Stakeholders' involvement in the PrevHPV intervention development process.

Professional stakeholders participating in the working group	Other stakeholders involved in the intervention development process
*Component 1: Adolescents' and parents' education and motivation*	
Expert in education and health promotion (*n* = 1) Expert in public health, responsible for medical students' training (*n* = 1) Expert in education sciences (*n* = 2) School nurse technical advisor at the school district level (*n* = 1) Expert in serious video games' development (*n* = 2)	*Serious video game*: adolescents and parents of adolescents (*n* = 17) provided feedback on the serious video game's visual aspects, suitability and readability of the quizzes (questions, answers) through online questionnaires (March–April 2020). *Videos*: one medical student created the videos as part of a contest organised by the research teams (April–October 2020). *School staff handbook*: one teacher in life sciences and one school nurse technical advisor critically revised the handbook which describes activities to implement during sessions with adolescents (June 2021).
*Component 2: General practitioners' (GP) training*	
GP (*n* = 9)	*Decision aid tool—phase 1 (design)*: adolescents' knowledge, beliefs towards HPV and its vaccination, needs and expectations towards such a tool (e.g., content, visual aspects) were explored through online focus groups (*n* = 14 adolescents) (October–December 2020). *Decision aid tool—phase 2 (test of the alpha version)* Adolescents (*n* = 6) and parents of adolescents (*n* = 8) provided feedback on the decision aid tool through online focus groups and one individual interview (January–April 2021). *GPs* (*n* = 11) pilot tested the decision aid tool in real‐life settings during 2‐6 weeks and provided feedback to the research team through individual semistructured interviews (May–September 2021).
*Component 3: Easier access to vaccination*	
Medical staff from vaccination centres (*n* = 2) School nurse technical advisor at the school district level (*n* = 1) School psychologist (*n* = 1)	*Vaccination day poster*: adolescents (*n* = 5), one school nurse technical advisor at the school district level and one GP provided feedback on the poster (e.g., visual aspects, suitability) aimed at informing adolescents on the vaccination day in the school premises (June 2021).

Abbreviation: HPV, human papillomavirus.

During the development process of specific tools, other stakeholders (e.g., adolescents, parents of adolescents, health students) were involved to coproduce the tools and/or providing feedback on some features (e.g., length, suitability, readability, visual aspects) (see details in Table 1).

### 2.9 How the intervention changed in content and format from the start of the development process

Due to the iterative nature of the intervention development process, there were some changes in the intervention content and format throughout the development process.

Regarding the content, based on results from the PrevHPV diagnostic phase and discussions amongst working groups, we made special efforts to define the best way to communicate HPV and its vaccination amongst adolescents and their parents. For example, we presented HPV infection as a sexually transmitted infection, and have been careful to talk about cancer risks without inducing fear. Also, during the development of an eHealth tool targeting adolescents (a serious video game, see details below in Section 3.1) feedback from parents and adolescents also led to several changes to improve readability and suitability; minor changes included changing a word to an easier one or rewording some questions/answers that were hard to understand.

Regarding the format, a noticeable change was in the mode of delivery of the information action targeting parents of adolescents. We initially planned to organise face‐to‐face meetings on school premises. Due to the COVID‐19 pandemic, we switched to online meetings.

## 3 RESULTS

The three components of the PrevHPV intervention are described below using the Template for Intervention Description and Replication checklist as a guide.^58^ For more details, see the completed checklist in Supporting Information Materials: Appendix D.

### 3.1 Adolescents and parents' education and motivation (component 1)

This component is carried out in middle schools because schools occupy a great part of adolescents' life and offer a unique opportunity to reach most adolescents.

First, component 1 includes an online information group session (duration: 1 h 30 min) on HPV infection and vaccination for parents of adolescents attending middle schools. The web conference was delivered by two medical experts on HPV, using a standardised presentation. A discussion is opened for parents' questions and/or comments at half‐time and at the end of the session. Parents can also access a replay of the web conference and additional information resources on HPV and its vaccination on an internet website developed as part of the project.

Second, adolescents from middle schools participate during school hours in two educational group sessions on HPV infections and vaccination, using a pedagogy based on active learning. These sessions (duration: 2 h each) are delivered by the school staff (e.g., nurses, teachers in life sciences) using an educational package comprising:

1. A handbook that describes activities to be implemented during each session. Each session consists of three activities based on eHealth tools (videos, serious video games), discussions/debates or role‐playing to motivate the active and interactive participation of pupils. Between the two sessions, adolescents are invited to carry out a small investigation of knowledge and attitudes about HPV infection and vaccination amongst some of their relatives (see Supporting Information Materials: Appendix E, for more details);
2. Six short videos (12 min in total) and a fact sheet created by a medical student and
3. A serious video game accessible on an Internet website were developed as part of the project. This tool is a digital game applied to educate pupils on HPV infection and vaccination and is not primarily intended for entertainment purposes.^49^

Before the sessions, the school staff is encouraged to attend an e‐learning training course developed as part of the project using the Wooclap platform. This includes presentations (e.g., on HPV infections, vaccinations, cervical cancer) and some quizzes (duration: 1 h).

### 3.2 GPs' training (component 2)

Component 2 consists of an individual e‐learning training session for GPs i.e. accessible on computers and smartphones. Lasting 3 h, GPs are able to access the training whenever they want and then progress at their own pace.

The training includes 12 videos divided into three main parts:

1. Up‐to‐date information on HPV infections and vaccination (vaccine coverage, safety and efficacy);
2. An introduction to the use of motivational interviewing techniques in the field of vaccination (theory and practice through role‐playing) and
3. A presentation of a decision aid tool developed as part of the intervention and explanations on how to use it during consultations. This tool, developed in accordance with the International Patient Decision Aid Standards,^59^, ^60^ aims at supporting hesitant parents/adolescents by making their decision about HPV vaccination explicit, providing information about options and associated benefits/harms, and helping clarify congruence between decisions and personal values.

### 3.3 Easier access to HPV vaccination (component 3)

Component 3 consists of one or several (depending on the number of parental consents returned) vaccination day(s) on the school premises during which health professionals (e.g., one physician and one nurse) from the local vaccination centre initiate HPV vaccination in accordance with safety and hygiene standards. Vaccination with the nonavalent HPV vaccine is offered free of charge without any prior medical prescription.

Before the vaccination day, school staff provide parents with information sheets and consent forms and then collect parents' written consents. They are also encouraged to display posters aimed at informing pupils about the vaccination day on school premises.

During the vaccination day, health professionals from the vaccination centre check the adolescents’ eligibility for vaccination (i.e., ≥11 years old, never vaccinated against HPV, with no contraindication to vaccination, and whose parents have given their written consent). They provide each vaccinated adolescent with several documents: a medical prescription for the second injection which will be performed by the adolescent's GP (or another health professional allowed to vaccinate against HPV) and a letter to inform her/him about the initiation of the HPV vaccination; a letter to his or her parents to confirm that he/she has been vaccinated and remind them of the vaccination schedule; and a letter to the pharmacist to inform her/him about the initiation of the vaccination.

## 4 DISCUSSION

In this paper, we described the development process of the PrevHPV school‐based and primary care‐based multicomponent intervention whose primary aim was to improve HPV vaccine coverage amongst French adolescents.

We described its development in a transparent and structured manner using the GUIDED checklist as recommended by the latest UK MRC framework for the development and evaluation of complex interventions.^61^ This approach helps intervention developers/funders understand the context and methods that were used and make judgements about the quality and relevance of the intervention and whether to implement an intervention within their specific context. It also enables methodological lessons to be learned and incorporated into future intervention development studies.^19^

The PrevHPV intervention development process has several strengths. We used both published research evidence and results from the PrevHPV diagnostic phase on target populations' needs to develop a multicomponent intervention that addresses a range of barriers and enablers of HPV vaccination. It is in line with the behaviour change model ‘Capability, Opportunity and Motivation model of Behaviour’ which argues that three key components interact to generate behaviour: Capability (knowledge and skills), Opportunity (physical and social), and Motivation (reflective and automatic).^62^, ^63^ Besides, we used a participatory approach in a co‐construction process involving adolescents, parents, GPs, staff from schools and vaccination centres in the activities/tools development.^57^ We also involved regional and national stakeholders (e.g., policymakers, funders) throughout the development process. We have also systematically paid attention to the future implementation of the intervention in a real‐world context. This approach is recommended to develop new interventions that have a better chance of being effective when evaluated and then of being adopted widely in the real world.^64^ One limitation of the intervention development process is that it was conducted during the COVID‐19 pandemic. As schools were closed from March to May 2020 in France, the collection of primary data (the PrevHPV diagnostic phase) had to be delayed. The pandemic context has also limited the availability of stakeholders and the opportunities to involve them in face‐to‐face interviews/meetings. This has finally required great adaptability from all professionals involved in the development process to maintain collaborative work through online meetings. In addition, the stakeholders involved in the development process were volunteered and thus probably particularly interested in the topic and supportive of the HPV vaccination. It would have been helpful to test the intervention tools amongst vaccine‐hesitant people as well.

At the end of the intervention development process, we have a good understanding of the rationale of the PrevHPV intervention and the underpinning evidence and theory. We provided professionals (e.g., school staff, experts, GPs) with guidelines and tools that they can apply with some flexibility to take into account the constraints and the schools/GPs' practises environment.^65^ However, uncertainties remain regarding its reach (regarding parents' participation in the web conference, adolescents' participation in the vaccination day at school, and GPs' participation in the e‐learning training), dose and fidelity (regarding the two 2‐h sessions for adolescents). Results from the evaluation of the effectiveness and implementation of the PrevHPV intervention^18^ will help refine the intervention before, if efficient, scaling it up.

## 5 CONCLUSION

This paper uses the GUIDED checklist to describe the development process of the PrevHPV school‐based and primary care‐based multicomponent intervention aimed at improving HPV vaccine coverage amongst French adolescents. The next step is to build on the results of the evaluation of the PrevHPV intervention^18^ to refine it before providing tools and recommendations for a nationwide scale‐up.

## AUTHOR CONTRIBUTIONS

Morgane Michel, Anne‐Sophie Le Duc‐Banaszuk, Karine Chevreul, Aurélie Gauchet, Bruno Giraudeau, Judith E. Mueller, Amandine Gagneux‐Brunon and Nathalie Thilly conceived the protocol of the PrevHPV Programme. Amandine Gagneux‐Brunon led the development of the adolescents and parents' education and motivation component with input from Aurélie Bocquier, Sébastien Bruel, Stéphanie Bonnay, Marion Branchereau, Sandra Chyderiotis, Aurélie Gauchet, Judith E. Mueller and Nathalie Thilly. Sébastien Bruel and Dragos‐Paul Hagiu led the development of the decision aid tool and contributed to the development of the general practitioners' training component. Anne‐Sophie Le Duc‐Banaszuk and Marion Branchereau led the development of access to vaccination in the school component with input from Aurélie Bocquier, Stéphanie Bonnay, Aurélie Gauchet and Amandine Gagneux‐Brunon. Stéphanie Bonnay facilitated the partnership between the teams of the consortium and with the steering committee. Aurélie Bocquier and Nathalie Thilly drafted the first version of the manuscript and all authors provided comments and feedback for improvement of the manuscript. All authors approved the final version of the manuscript.

## CONFLICT OF INTEREST STATEMENT

The authors declare no conflict of interest.

## ETHICS STATEMENT

The PrevHPV diagnostic phase was granted approval by the Evaluation Committee of Inserm, the Institutional Review Board (IRB00003888, IORG0003254, FWA00005831) on 10 December 2019. All study participants gave their informed nonopposition to participation, in line with French legal guidelines.

## Supporting information

Supporting information.

## Data Availability

The data that support the findings of the PrevHPV diagnostic phase are available from the French National Institute for Health and Medical Research (Inserm) but restrictions apply to the availability of these data, which are not publicly available. Data are, however, available from the authors upon reasonable request and with permission of the Inserm. The reuse of data is subject to compliance with the General Data Protection Regulation and French regulations.
