# Supplementary material for: Co‐development of a school‐based and primary care‐based multicomponent intervention to improve HPV vaccine coverage amongst French adolescents (the PrevHPV Study)
Source: Health Expect. 2023 Jun 13;26(5):1843–53. doi: 10.1111/hex.13778 (PMC10485335; doi:10.1111/hex.13778)
Supplement: Supplementary file 1 — Supporting information. [file HEX-26--s001.docx]

**Supplementary Materials (online)**

**Appendix A.** Teams conducting the PrevHPV programme (The PrevHPV Consortium)

| **Team n°** | **Contact/scientific leader** | **Field of expertise** |
| --- | --- | --- |
| **1** | EA 4360 APEMAC - Université de Lorraine  9 av. de la Forêt de Haye - BP 20199 - 54505 VANDOEUVRE LES NANCY Cedex  Scientific leader, principal investigator: Pr THILLY Nathalie (email: [n.thilly@chru-nancy.fr](mailto:n.thilly@chru-nancy.fr)) | Epidemiology and Public health |
| **2** | Département de Médecine Générale - Université Paris Cité - 24 rue du Faubourg Saint-Jacques -75679 PARIS Cedex 14  Scientific leader: Pr GILBERG Serge (email: [sergegilberg@gmail.com](mailto:sergegilberg@gmail.com)) | Primary Care |
| **3** | Laboratoire Interuniversitaire de Psychologie - UFR Sciences de l'Homme et de la Société - Université Grenoble Alpes  BP 47 - 38040 GRENOBLE Cedex 9  Scientific leader: Dr GAUCHET Aurélie (email: [aurelie.gauchet@univ-grenoble-alpes.fr](mailto:aurelie.gauchet@univ-grenoble-alpes.fr)) | Health Psychology |
| **4** | CRCDC Pays de la Loire  5 rue des Basses Fouassières - 49000 ANGERS  Scientific leader: Dr LE DUC-BANASZUK Anne-Sophie (email: [as.banaszuk@depistagecancers.fr](mailto:as.banaszuk@depistagecancers.fr)) | Public Health, Cancer prevention |
| **5** | Campus Santé Innovations - Faculté de Médecine Jacques Lisfranc 10 rue de la Marandière - 42270 SAINT-PRIEST-EN-JAREZ  Scientific leader: Dr GAGNEUX-BRUNON Amandine (email: [amandine.gagneux-brunon@chu-st-etienne.fr](mailto:amandine.gagneux-brunon@chu-st-etienne.fr)) | Infectious Diseases |
| **6** | INSERM UMR 1123 ECEVE, Université Paris Cité,75010 PARIS  Scientific leader: Pr CHEVREUL Karine (email: [karine.chevreul@inserm.fr](mailto:karine.chevreul@inserm.fr)) | Health Economics |
| **7** | Institut Pasteur - 25 rue du Dr Roux - 75724 Paris cedex 15  Scientific leader: Dr MUELLER Judith (email: judith.mueller@ehesp.fr) | Epidemiology and Public health |
| **8** | CHRU de Tours - Centre d’investigation Clinique Bretonneau - 37044 Tours cedex 9  Scientific leader: Pr GIRAUDEAU Bruno (email: [bruno.giraudeau@univ-tours.fr](mailto:bruno.giraudeau@univ-tours.fr)) | Biostatistics |

**Appendix B.** GUIDED checklist (a guideline for reporting for intervention development studies)

| Item description | Explanation | Page in manuscript where item is located | Other* |
| --- | --- | --- | --- |
| 1.Report the context for which the intervention was developed. | Understanding the context in which an intervention was developed informs readers about the suitability and transferability of the intervention to the context in which they are considering evaluating, adapting or using the intervention. Context here can include place, organisational and wider sociopolitical factors that may influence the development and/or delivery of the intervention (15). | Subsection 2.1 | N/A |
| 2.Report the purpose of the intervention development process. | Clearly describing the purpose of the intervention specifies what it sets out to achieve. The purpose may be informed by research priorities, for example those identified in systematic reviews, evidence gaps set out in practice guidance such as The National Institute for Health and Care Excellence or specific prioritisation exercises such as those undertaken with patients and practitioners through the James Lind Alliance. | Subsection 2.2 | N/A |
| 3. Report the target population for the intervention development process. | The target population is the population that will potentially benefit from the intervention – this may include patients, clinicians, and/or members of the public. If the target population is clearly described then readers will be able to understand the relevance of the intervention to their own research or practice. Health inequalities, gender and ethnicity are features of the target population that may be relevant to intervention development processes. | Subsection 2.3 | N/A |
| 4. Report how any published intervention development approach contributed to the development process | Many formal intervention development approaches exist and are used to guide the intervention development process (e.g. 6Squid (16) or The Person Based Approach to Intervention Development (17)). Where a formal intervention development approach is used, it is helpful to describe the process that was followed, including any deviations. More general approaches to intervention development also exist and have been categorised as follows (3):- Target Population-centred intervention development; evidence and theory-based intervention development; partnership intervention development; implementation-based intervention development; efficacybased intervention development; step or phased-based intervention development; and intervention-specific intervention development (3). These approaches do not always have specific guidance that describe their use. Nevertheless, it is helpful to give a rich description of how any published approach was operationalised | Subsection 2.4 | N/A |
| 5. Report how evidence from different sources informed the intervention development process. | Intervention development is often based on published evidence and/or primary data that has been collected to inform the intervention development process. It is useful to describe and reference all forms of evidence and data that have informed the development of the intervention because evidence bases can change rapidly, and to explain the manner in which the evidence and/or data was used. Understanding what evidence was and was not available at the time of intervention development can help readers to assess transferability to their current situation. | Subsection 2.5 | N/A |
| 6. Report how/if published theory informed the intervention development process. | Reporting whether and how theory informed the intervention development process aids the reader’s understanding of the theoretical rationale that underpins the intervention. Though not mentioned in the e-Delphi or consensus meeting, it became increasingly apparent through the development of our guidance that this theory item could relate to either existing published theory or programme theory | Subsection 2.6 | N/A |
| 7. Report any use of components from an existing intervention in the current intervention development process. | Some interventions are developed with components that have been adopted from existing interventions. Clearly identifying components that have been adopted or adapted and acknowledging their original source helps the reader to understand and distinguish between the novel and adopted components of the new intervention. | N/A | N/A |
| 8. Report any guiding principles, people or factors that were prioritised when making decisions during the intervention development process. | Reporting any guiding principles that governed the development of the application helps the reader to understand the authors’ reasoning behind the decisions that were made. These could include the examples of particular populations who views are being considered when designing the intervention, the modality that is viewed as being most appropriate, design features considered important for the target population, or the potential for the intervention to be scaled up. | Subsection 2.7 | N/A |
| 9. Report how stakeholders contributed to the intervention development process. | Potential stakeholders can include patient and community representatives, local and national policy makers, health care providers and those paying for or commissioning health care. Each of these groups may influence the intervention development process in different ways. Specifying how differing groups of stakeholders contributed to the intervention development process helps the reader to understand how stakeholders were involved and the degree of influence they had on the overall process. Further detail on how to integrate stakeholder contributions within intervention reporting are available (19). | Subsection 2.8 | N/A |
| 10. Report how the intervention changed in content and format from the start of the intervention development process. | Intervention development is frequently an iterative process. The conclusion of the initial phase of intervention development does not necessarily mean that all uncertainties have been addressed. It is helpful to list remaining uncertainties such as the intervention intensity, mode of delivery, materials, procedures, or type of location that the intervention is most suitable for. This can guide other researchers to potential future areas of research and practitioners about uncertainties relevant to their healthcare context. | Subsection 2.9 | N/A |
| 11. Report any changes to interventions required or likely to be required for subgroups. | Specifying any changes that the intervention development team perceive are required for the intervention to be delivered or tailored to specific sub groups enables readers to understand the applicability of the intervention to their target population or context. These changes could include changes to personnel delivering the intervention, to the content of the intervention, or to the mode of delivery of the intervention. | N/A | N/A |
| 12. Report important uncertainties at the end of the intervention development process | Intervention development is frequently an iterative process. The conclusion of the initial phase of intervention development does not necessarily mean that all uncertainties have been addressed. It is helpful to list remaining uncertainties such as the intervention intensity, mode of delivery, materials, procedures, or type of location that the intervention is most suitable for. This can guide other researchers to potential future areas of research and practitioners about uncertainties relevant to their healthcare context. | Discussion | N/A |
| 13. Follow TIDieR guidance when describing the developed intervention. | Interventions have been poorly reported for a number of years. In response to this, internationally recognized guidance has been published to support the high quality reporting of health care? interventions5and public health interventions14. This guidance should therefore be followed when describing a developed intervention. | Results and Appendix D | N/A |
| 14. Report the intervention development process in an open access format. | Unless reports of intervention development are available people considering using an intervention cannot understand the process that was undertaken and make a judgement about its appropriateness to their context. It also limits cumulative learning about intervention development methodology and observed consequences at later evaluation, translation and implementation stages. Reporting intervention development in an open access (Gold or Green) publishing format increases the accessibility and visibility of intervention development research and makes it more likely to be read and used. Potential platforms for open access publication of intervention development include open access journal publications, freely accessible funder reports or a study web-page that details the intervention development process. | The present article | N/A |

*e.g. if item is reported elsewhere, then the location of this information can be stated here.

**Appendix C.** Data collection performed as part of the diagnostic phase of the PrevHPV Program

| **Target population** | **Type of study** | | | |
| --- | --- | --- | --- | --- |
|  | **Discrete choice experiment (online)** | **Quantitative cross-sectional survey (online)** | **Focus groups** | **Semi-structured individual interviews** |
| **Adolescents^a^** | n = 1458 participants | n = 827 participants | **/** | **/** |
| **Parents of adolescents^b^** | n = 1291 participants | **/** | 4 focus groups  (n = 15 parents in total) | **/** |
| **School staff^c^** (heads of schools, teachers, school nurses, support staff) | **/** | n = 280 participants | 3 focus groups  (n = 14 participants in total) | **/** |
| **General practitioners** | **/** | **/** | **/** | n = 26 participants |
| **Health students** | **/** | n = 596 participants | **/** | **/** |

^a^ Adolescents typically aged 13-15 years, corresponding to grades 8 and 9 in the US educational system.

^b^ Parents of adolescents typically aged 11-15 years, corresponding to grades 6-9 in the US educational system.

^c^ School staff from middle schools (pupils typically aged 11-15 years, corresponding to grades 6-9 in the US educational system).

**Appendix D.** TIDieR Checklist for the PrevHPV multicomponent intervention

| **Item number** | **Item** | **Description** |
| --- | --- | --- |
| BRIEF NAME | | |
| 1. | Provide the name or a phrase that describes the intervention. | A School-based and Primary Care-based Multicomponent Intervention to improve Human papillomavirus (HPV) Vaccination Acceptability (PrevHPV) |
| WHY | | |
| 2. | Describe any rationale, theory, or goal of the elements essential to the intervention. | Vaccination is an effective and safe strategy to prevent Human papillomavirus (HPV) infection and related harms. Despite various efforts by French authorities to improve HPV vaccine coverage these past few years, vaccine coverage has remained far lower than in most other high income countries. To improve it, we have coconstructed with stakeholders a school-based and primary care-based multicomponent intervention.  Rational for component 1 “Adolescents and parents’ education and motivation”:  Lack of knowledge on HPV infection and concerns about vaccine effectiveness and safety are described as being strong barriers to HPV vaccination (Karafillakis et al., 2019; Rodriguez et al., 2020). These elements have also been found in surveys performed during the diagnostic phase of the PrevHPV Program. Interventions targeting parents and adolescents’ psychosocial factors (knowledge, beliefs, outcome expectations, intention to vaccinate) have shown promising results abroad (Jacobson et al., 2016; Rodriguez et al., 2019; Smulian et al., 2016; Vollrath et al., 2018; Walling et al., 2016), and eHealth tools (e.g., videos, websites, serious video games) are encouraged (Dumit et al., 2018b; Ohannessian et al., 2016), as well as participatory learning (Simovska, 2011).  Rational for component 2 “GPs’ training”:  Healthcare providers, especially general practitioners (GPs), have a fundamental role in parents and adolescents’ decision-making process towards HPV vaccination (Rodriguez et al., 2020). However, they face difficulties in informing about HPV vaccination and convincing hesitant patients to get vaccinated for HPV (Bouchez et al., 2021). Educational strategies based on motivational interview (Gagneur et al., 2019) and decision aids (Dempsey et al., 2018; Stacey et al., 2017) have been found to be promising ways to motivate hesitant patients to accept vaccination. Decision aid tools support patients by making their decisions explicit, providing information about options and associated benefits/harms, and helping clarify congruence between decisions and personal values (Dempsey et al., 2018; Stacey et al., 2017).  Rational for component 3 “Easier access to HPV vaccination”:  Organisational and financial barriers to HPV vaccination have been identified in France (Nguyen-Huu et al., 2020), and probably limit the HPV vaccine coverage. Care pathway to access vaccination is often complex: for the majority of cases, adolescents and their parents must make an appointment with a physician to get the vaccine prescription, then go to a community pharmacy to obtain the vaccine, and finally make another appointment with their physician for its administration. Occasionally, individuals will also get vaccinated at hospital vaccination centres, but their geographical accessibility can be difficult. In addition, HPV vaccine is only partially reimbursed by the French national Health Insurance, and some patients may be charged out-of-pocket costs (Nguyen-Huu et al., 2020). Most European countries with high HPV vaccine coverage such as Belgium Flanders, the UK and Scandinavian countries have implemented school-based vaccination programmes with no mandatory medical prescription. |
| WHAT | | |
| 3. | Materials: Describe any physical or informational materials used in the intervention, including those provided to participants or used in intervention delivery or in training of intervention providers. Provide information on where the materials can be accessed (e.g. online appendix, URL). | Materials provided for component 1 “Adolescents and parents’ education and motivation”:  - Webconference for parents: a standardized presentation presented by medical experts.  - Educational group sessions for adolescents: a “school staff handbook” that describes activities that should be implemented during each session; six short videos and a fact sheet; a serious video game accessible on an internet website; an e-learning training for school staff.  Materials provided for component 2 “GPs’ training”:  An e-learning training session including 12 videos divided in three main parts: (1) updated information on HPV infections and vaccination (vaccine coverage, safety, efficacy); (2) an introduction to the use of motivational interviewing techniques in the field of vaccination (theory and practice through role-playing); (3) a presentation of the decision aid tool developed as part of the intervention and explanation of how to use it during consultations.  Materials provided for component 3 “Easier access to HPV vaccination”:  Parents information sheets and consent forms; posters to inform pupils about the vaccination day; letter templates to inform vaccinated adolescents’ parents, general practitioner and pharmacist. |
| 4. | Procedures: Describe each of the procedures, activities, and/or processes used in the intervention, including any enabling or support activities. | Procedures for component 1 “Adolescents and parents’ education and motivation”:  - Project leader: each participating middle school designate a “project leader” (e.g., school nurse) in charge of coordinating all actions implemented in the school. The research team provided her/him a “project leader handbook” describing all actions to implement and the timeline.  - Webconference for parents: school staff invite parents through email or any other usual communication channels used in middle schools. The email informs parents about the context, objective and content of the webconference and provides them with practical details and the link of the webconference. During the webconference, parents can ask questions trough the chat (e.g., using Zoom platform).  - Educational group sessions for adolescents: the “project leader” inform all school staff about the organization of these sessions to identify volunteers. School staff organized the implementation of these sessions in pupils’ schedules. School staff can access an e-learning training (using Wooclap platform).  Procedures for component 2 “GPs’ training”:  GPs who accepted to attend the training received practical information from the research team to access the e-learning platform (using LearnyBox platform). GPs can access the e-learning training whenever they want.  Procedures for component 3 “Easier access to HPV vaccination”:  The research team provided the health professionals from the vaccination center a “vaccination center handbook” describing all actions to implement as part of the vaccination day and the timeline. Before the vaccination day, the middle school’s project leader and the professionals from the vaccination centers were encouraged to talk together to organize the practical aspects of the action (e.g., rooms and material needed/available, schedule). |
| WHO PROVIDED | | |
| 5. | For each category of intervention provider (e.g. psychologist, nursing assistant), describe their expertise, background and any specific training given. | Component 1 “Adolescents and parents’ education and motivation”:  - Webconference for parents: two medical experts (e.g., infectious disease specialist, gynecologist, general practitioner).  - Educational group sessions for adolescents: school nurse, teacher in life sciences or any other teacher or school staff trained on emotional and sexual life. School staff are encouraged to attend an e-learning training developed as part of the project.  Component 2 “GPs’ training”:  GPs who attended an e-learning training session including 12 videos divided in three main parts: (1) updated information on HPV infections and vaccination (vaccine coverage, safety, efficacy); (2) an introduction to the use of motivational interviewing techniques in the field of vaccination (theory and practice through role-playing); (3) a presentation of the decision aid tool developed as part of the intervention and explanation of how to use it during consultations.  Component 3 “Easier access to HPV vaccination”:  Professionals from the local vaccination center (e.g., one physician and one nurse). |
| HOW | | |
| 6. | Describe the modes of delivery (e.g. face-to-face or by some other mechanism, such as internet or telephone) of the intervention and whether it was provided individually or in a group. | Component 1 “Adolescents and parents’ education and motivation”:  - Webconference for parents: online, provided in a group.  - Educational group sessions for adolescents: face-to-face, provided in a group. Tools/activities used during these sessions: serious video game (online, provided individually), videos (online, provided in a group), participatory learning activities (face-to-face, provided in a group).  Component 2 “GPs’ training”:  Online, provided individually.  Materials provided for component 3 “Easier access to HPV vaccination”:  Face-to-face, provided individually. |
| WHERE | | |
| 7. | Describe the type(s) of location(s) where the intervention occurred, including any necessary infrastructure or relevant features. | Component 1 “Adolescents and parents’ education and motivation”:  - Webconference for parents: online.  - Educational group sessions for adolescents: in middle school premises. Due to routine practices about sexuality education and the life science curriculum in France, groups sessions were organized for adolescents aged 13-15 years.  Component 2 “GPs’ training”:  Online.  Materials provided for component 3 “Easier access to HPV vaccination”:  In middle school premises. |
| WHEN and HOW MUCH | | |
| 8. | Describe the number of times the intervention was delivered and over what period of time including the number of sessions, their schedule, and their duration, intensity or dose. | Component 1 “Adolescents and parents’ education and motivation”:  - Webconference for parents: once (duration: 1h30); two time slots were offered to each parent.  - Educational group sessions for adolescents: two sessions (duration: 2h each) over a 2-month period.  Component 2 “GPs’ training”:  E-learning training session (duration: 3 hours) accessible whenever GPs want.  Component 3 “Easier access to HPV vaccination”:  One vaccination day (or more depending on the number of adolescents to vaccine). |
| TAILORING | | |
| 9. | If the intervention was planned to be personalised, titrated or adapted, then describe what, why, when, and how. | We provided professionals with guidelines and tools that they can apply with some flexibility to take into account the constraints and the schools/GPs’ practices. |
| MODIFICATIONS | | |
| 10. | If the intervention was modified during the course of the study, describe the changes (what, why, when, and how). | N/A - Adaptations will be assessed as part of the implementation evaluation during the PrevHPV study (Bocquier et al., 2022). |
| HOW WELL | | |
| 11. | Planned: If intervention adherence or fidelity was assessed, describe how and by whom, and if any strategies were used to maintain or improve fidelity, describe them. | Intervention components’ dose and fidelity will be assessed as part of the implementation evaluation during the PrevHPV study (Bocquier et al., 2022). They will be assessed using regular activity reports collected on a standardized form during components’ implementation completed by members of the research teams as well as school staff and health professionals from vaccination centers. |
| 12. | Actual: If intervention adherence or fidelity was assessed, describe the extent to which the intervention was delivered as planned. | N/A - Intervention fidelity will be assessed as part of the implementation evaluation during the PrevHPV study (Bocquier et al., 2022). |

**References**

Bocquier, A., Michel, M., Giraudeau, B., Bonnay, S., Gagneux-Brunon, A., Gauchet, A., Gilberg, S., Le Duc-Banaszuk, A.-S., Mueller, J.E., Chevreul, K., Thilly, N., 2022. Impact of a school-based and primary care-based multicomponent intervention on HPV vaccination coverage among French adolescents: a cluster randomised controlled trial protocol (the PrevHPV study). BMJ Open 12, e057943. https://doi.org/10.1136/bmjopen-2021-057943

Bouchez, M., Ward, J.K., Bocquier, A., Benamouzig, D., Peretti-Watel, P., Seror, V., Verger, P., 2021. Physicians’ decision processes about the HPV vaccine: A qualitative study. Vaccine 39, 521–528. https://doi.org/10.1016/j.vaccine.2020.12.019

Dempsey, A.F., Pyrznawoski, J., Lockhart, S., Barnard, J., Campagna, E.J., Garrett, K., Fisher, A., Dickinson, L.M., O’Leary, S.T., 2018. Effect of a Health Care Professional Communication Training Intervention on Adolescent Human Papillomavirus Vaccination: A Cluster Randomized Clinical Trial. JAMA Pediatr 172, e180016. https://doi.org/10.1001/jamapediatrics.2018.0016

Dumit, E.M., Novillo-Ortiz, D., Contreras, M., Velandia, M., Danovaro-Holliday, M.C., 2018a. The use of eHealth with immunizations: An overview of systematic reviews. Vaccine 36, 7923–7928. https://doi.org/10.1016/j.vaccine.2018.06.076

Gagneur, A., Battista, M.-C., Boucher, F.D., Tapiero, B., Quach, C., De Wals, P., Lemaitre, T., Farrands, A., Boulianne, N., Sauvageau, C., Ouakki, M., Gosselin, V., Petit, G., Jacques, M.-C., Dubé, È., 2019. Promoting vaccination in maternity wards ─ motivational interview technique reduces hesitancy and enhances intention to vaccinate, results from a multicentre non-controlled pre- and post-intervention RCT-nested study, Quebec, March 2014 to February 2015. Euro Surveill 24, 1800641. https://doi.org/10.2807/1560-7917.ES.2019.24.36.1800641

Jacobson, R.M., Agunwamba, A.A., St. Sauver, J.L., Finney Rutten, L.J., 2016. The most effective and promising population health strategies to advance human papillomavirus vaccination. Expert Rev Vaccines 15, 257–269. https://doi.org/10.1586/14760584.2016.1116947

Karafillakis, E., Simas, C., Jarrett, C., Verger, P., Peretti-Watel, P., Dib, F., De Angelis, S., Takacs, J., Ali, K.A., Pastore Celentano, L., Larson, H., 2019. HPV vaccination in a context of public mistrust and uncertainty: a systematic literature review of determinants of HPV vaccine hesitancy in Europe. Hum Vaccin Immunother 15, 1615–1627. https://doi.org/10.1080/21645515.2018.1564436

Nguyen-Huu, N.-H., Thilly, N., Derrough, T., Sdona, E., Claudot, F., Pulcini, C., Agrinier, N., 2020. Human papillomavirus vaccination coverage, policies, and practical implementation across Europe. Vaccine 38, 1315–1331. https://doi.org/10.1016/j.vaccine.2019.11.081

Ohannessian, R., Yaghobian, S., Verger, P., Vanhems, P., 2016. A systematic review of serious video games used for vaccination. Vaccine 34, 4478–4483. https://doi.org/10.1016/j.vaccine.2016.07.048

Rodriguez, A.M., Do, T.Q.N., Goodman, M., Schmeler, K.M., Kaul, S., Kuo, Y.-F., 2019. Human Papillomavirus Vaccine Interventions in the U.S.: A Systematic Review and Meta-analysis. Am J Prev Med 56, 591–602. https://doi.org/10.1016/j.amepre.2018.10.033

Rodriguez, S.A., Mullen, P.D., Lopez, D.M., Savas, L.S., Fernández, M.E., 2020. Factors associated with adolescent HPV vaccination in the U.S.: A systematic review of reviews and multilevel framework to inform intervention development. Prev Med 131, 105968. https://doi.org/10.1016/j.ypmed.2019.105968

Simovska, V., 2011. Case Study of a Participatory Health Promotion Intervention in School. Democracy and Education 20, Article 4.

Smulian, E.A., Mitchell, K.R., Stokley, S., 2016. Interventions to increase HPV vaccination coverage: A systematic review. Hum Vaccin Immunother 12, 1566–1588. https://doi.org/10.1080/21645515.2015.1125055

Stacey, D., Légaré, F., Lewis, K., Barry, M.J., Bennett, C.L., Eden, K.B., Holmes-Rovner, M., Llewellyn-Thomas, H., Lyddiatt, A., Thomson, R., Trevena, L., 2017. Decision aids for people facing health treatment or screening decisions. Cochrane Database Syst Rev 4, CD001431. https://doi.org/10.1002/14651858.CD001431.pub5

Vollrath, K., Thul, S., Holcombe, J., 2018. Meaningful Methods for Increasing Human Papillomavirus Vaccination Rates: An Integrative Literature Review. J Pediatr Health Care 32, 119–132. https://doi.org/10.1016/j.pedhc.2017.07.005

Walling, E.B., Benzoni, N., Dornfeld, J., Bhandari, R., Sisk, B.A., Garbutt, J., Colditz, G., 2016. Interventions to Improve HPV Vaccine Uptake: A Systematic Review. Pediatrics 138. https://doi.org/10.1542/peds.2015-3863

**Appendix E.** Overview of the objectives and content of the two educational group sessions for adolescents proposed in the school staff handbook^a^

**First Session – What are “Human papillomaviruses”?**

**Objectives:** the purpose of this session is to introduce the PrevHPV project and to inform pupils in a participatory and interactive way about HPV and vaccination. At the end of this session, pupils will:

- Have learned about the objectives of the PrevHPV project,

- Have obtained clear and evidence-based information about HPV and vaccination,

- Have expressed their perceptions of HPV and vaccination,

- Be aware of some “false” beliefs about HPV and vaccination.

**Duration:** 2 hours.

**Content:** 3 activities and, at the end of the session, a synthesis of the elements discussed.

| **Activity #1 – The PrevHPV project** | | | 20-30 min |
| --- | --- | --- | --- |
|  | ⌖ Presentation of the PrevHPV project. | ✀ Tool: a short presentation of the project. |  |
| **Activity #2 – Human papillomaviruses: Who are they? What do they do? Where do they go?** | | | 30 min |
|  | ⌖ Enable adolescents to learn about HPV and HPV vaccination using a participatory approach and some videos. | ✀ Tool: post-it (for brainstorming about the term papillomavirus) and 6 short videos. |  |
| **Activity #3 – And now let's play "HPV, in search of action"** | | | 30 min |
|  | ⌖ Enable adolescents to learn about HPV and HPV vaccination using a serious video game. | ✀ Tool: a serious video game. |  |
| **Synthesis of the session** | | | 20-30 min |
|  | ⌖ Answer pupils’ questions.  ⌖ Introduce the second session and present the small survey to be carried out between the 2 sessions. | ✀ Tool: the questionnaire for the small survey about HPV and vaccination. |  |

**Second Session – Vaccination: what is it? What does it do?**

**Objectives:** the purpose of this session is to talk about some of the elements presented in the first session and to discuss HPV vaccination more in depth. At the end of this session, pupils will:

- Be aware of the different representations that exist among the general population on HPV and vaccination,

- Have acquired additional knowledge on vaccination,

- Know that vaccination is the only effective way to prevent HPV infections,

- Will have thought about their own reasons for getting vaccinated or not.

**Duration:** 2 hours.

**Content:** 3 activities and, at the end of the session, a synthesis of the elements discussed.

| **Activity #1 – The surveys’ results** | | | 20-30 min |
| --- | --- | --- | --- |
|  | ⌖ Become aware of the existing representations on HPV and vaccination in the population and deconstruct erroneous ideas / false beliefs. | ✀ Tool: results from the small survey, slides on vaccination. |  |
| **Activity #2 – What do you think about vaccination?** | | | 30 min |
|  | ⌖ Review the concepts acquired during the first session.  ⌖ Deconstruct adolescents’ false ideas and representations and reinforce the correct ones. | ✀ Tool: a list of statements on preconceived ideas about vaccination to be discussed with pupils in a participatory approach (“abaque de Régnier”). |  |
| **Activity #3 – Help Lucien: he is 14 years old, wants to ask his parents to get vaccinated against HPV but doesn't know how** | | | 30 min |
|  | ⌖ Review the concepts acquired during the first session.  ⌖ Learn about how to formulate arguments and to defend one’s choices. | ✀ Tool: scenario for the role playing activity. |  |
| **Synthesis of the session** | | | 20-30 min |
|  | ⌖ Answer pupils’ questions. | ✀ Tool: paper sheets and pencils (pupils write 1-3 questions about HPV or vaccination). |  |

^a^ The complete original version of the PrevHPV school staff handbook is available from the authors on request.
